# Review of machine perfusion studies in vascularized composite allotransplant preservation

**DOI:** 10.3389/frtra.2023.1323387

**Published:** 2023-12-20

**Authors:** Çağdaş Duru, Felor Biniazan, Nina Hadzimustafic, Andrew D'Elia, Valentina Shamoun, Siba Haykal

**Affiliations:** ^1^ Latner Thoracic Surgery Laboratories, University Health Network (UHN), Toronto, ON, Canada; ^2^ Temerty Faculty of Medicine, University of Toronto, Toronto, ON, Canada; ^3^ Plastic and Reconstructive Surgery, Temerty Faculty of Medicine, University of Toronto, Toronto, ON, Canada

**Keywords:** *ex-vivo* perfusion, *ex-vivo* limb perfusion, vascularized composite allograft (VCA), ischemia reperfusion (I/R) injury, hand transplantation

## Abstract

The applications of Vascularized composite allotransplantation (VCA) are increasing since the first successful hand transplantation in 1998. However, the abundance of muscle tissue makes VCA's vulnerable to ischemia-reperfusion injury (IRI), which has detrimental effects on the outcome of the procedure, restricting allowable donor-to-recipient time and limiting its widespread use. The current clinical method is Static cold storage (SCS) and this allows only 6 h before irreversible damage occurs upon reperfusion. In order to overcome this obstacle, the focus of research has been shifted towards the prospect of *ex-vivo* perfusion preservation which already has an established clinical role in solid organ transplants especially in the last decade. In this comprehensive qualitative review, we compile the literature on all VCA machine perfusion models and we aim to highlight the essentials of an *ex vivo* perfusion set-up, the different strategies, and their associated outcomes.

## Introduction

The history of organ preservation using machine perfusion dates back to the 1930s with the work of Carrell (1) and Lindbergh (2). The first successful clinical transplantation of machine-perfused donor organs was in the late 1960s with both the kidney (3) and liver (4) organs. However, this approach fell out of popularity for a period of time due to a better understanding of the benefits of cooling (5) and the development of preservation solutions (6–8) that provided an easier yet still effective method for organ preservation. This method is Static Cold Storage (SCS) and involves flushing the organ with a preservation solution and immersing it in the solution at 4°C. This allows up to 24-h preservation for kidneys (9) and around 12 h for livers (10, 11) without significant post-operative graft dysfunction.

To match the increasing need for organs, extended criteria donors (ECD) and donors after circulatory death (DCD) are used in increasing numbers (12, 13). These grafts are frail by definition, and machine perfusion systems come into play by allowing graft assessment and reconditioning, which cannot be achieved by SCS. For instance, *ex vivo* perfusion of lungs now allows high-risk organs or even discarded organs to be assessed and transplanted successfully with longer preservation times (14–16). Again, successful transplantation of declined or marginal livers can now be performed after resuscitation with machine perfusion (17, 18). Quality assessment of kidneys can also be performed before transplantation (19). Moreover, machine-perfused kidneys have been shown to have less frequent delayed graft function when compared to static cold preserved kidneys in the clinical setting (20). Machine perfusion systems now have an increasing clinical application in solid organ transplantations, exceeding the limits of SCS.

The relatively new field of vascularized composite tissue allotransplantation (VCA)—which is the simultaneous transfer of multiple types of tissues such as that of the skin, muscle, nerve, and bone as a single functioning unit—faces a distinct obstacle in terms of preservation. The obstacle is due to the abundance of muscle tissue that is highly metabolically active and sensitive to Ischemia-Reperfusion injury (IRI) (21, 22). IRI defines a series of well-studied predictable events that occur when the blood supply is cut in any given organ (23). In general terms, the depletion of ATP during ischemia results in the disruption of various membrane antiports (24), the mitochondrial electron transport chain (25), and various enzymes (26), resulting in acidosis and cellular swelling. Upon reperfusion, the accumulated cations continue to create an osmotic gradient after normalization of the extracellular space, which further induces cellular swelling and death (27). In addition, reactive oxygen species (ROS) reappear, and the reduced capability of the cell to withstand oxidative stress (28) again leads to cellular death, which clinically manifests as diminished function of the muscle (27). Also, the reperfusion phase initiates an inflammatory response (29), which is associated with sensitization and acute rejections (30). The SCS method here is not as successful as solid organs and allows only a period of 4–6 h before the detrimental effects of IRI are irreversible (27, 31).

In its third decade, the field of VCA is expanding, reaching over 140 hand–upper extremity transplantations (32) and 40 craniofacial transplantations (33) worldwide. As the clinical applications of VCA increase, and with respect to the shortcomings of SCS in muscle preservation, there has been a coincidental increase in the amount of research on the prospect of machine perfusion of limbs and other VCA models specifically in the last decade. This comprehensive review compiles the literature of all VCA machine perfusion models (Tables 1, 2) and aims to highlight the essentials of an *ex vivo* perfusion setup, the different strategies, and their associated outcomes.

**Table 1:** *Ex vivo* limb perfusion studies.

Author/Year	Animal model and groups	Temp.	PP Flow Total volume	Perfusate base	Additives	Duration (h)	Weight increase (%)	Reperfusion	Remarks
Pendexter et al. (34) 2023	Rat forelimbs and hindlimbs (protocol development)	21°C	Never exceed 35–40 mmHg	DMEM	Heparin	2	Actual numbers not given	No	Forelimbs mandate lower flow rates, 0.8 ml/min provides similar results to established hindlimb protocol.
	Hind limbs (*n* = 3) established protocol*		Flow adjusted to pressure	BSA 10%	Insulin				
	0.8 ml/min forelimbs (*n* = 4)			Dextran40	Dexamethasone				
	1.0 ml/min forelimbs (*n* = 4)			PEG35					
Meyers et al. (35) 2023	Swine forelimbs	38°C	90 mmHg	Plasmalyte (2 L)	Calcium gluconate	24 or	Hourly weight monitor	No	Weight gain precedes the decline of contractility and derangement of other physiological parameters and correlates with histological evidence of muscle injury.
	EVLP (*n* = 8)		Flow adjusted to pressure	Albumin	Sodium bicarbonate	>115 mmHg	2% at 13 ± 5 h		
	SCS (*n* = 8)		2.5 L Total circulating volume		Insulin	or >30 mmHg CP	5% at 15 ± 6 h		
					Cefazolin	or 20% drop O_2_	10% at 16 ± 6 h		MIS remained lower in all 6-h periods of SCS.
					Vancomycin	(20.5 ± 3.1)	20% at 19 ± 4 h		
					Methylprednisolone				
Burlage et al. (36) 2022	*Rat hind limb (three different solutions tested)	21°C	30–40 mmHg		Penicillin-Streptomycin	6		Transplantation	HBOC201 provides superior results vs. SCS and other perfusion forms.
	EVLP 1 (*n* = 4)		Flow adjusted to pressure	1. Muscle cell media + BSA	L-glutamine		48.8 (39.1–53.2)	30-day follow-up	
	EVLP 2 (*n* = 4)		500 ml	2. PEG added	Insulin		27.3 (20.5–41.6)		
	EVLP 3 (*n* = 4)			3. PEG&HBOC-201added	Heparin		4.9 (4.3–6.1)		After transplant, HBOC201 had the best survival on day 30.
	SCS (*n* = 4)				Hydrocortisone				
	Validation of protocol with transplant			With HBOC-201 group	Dexamethasone				
	EVLP (*n* = 13)								
	SCS-6 (*n* = 4)								
	SCS 24 (*n* = 5)								
	Untreated (*n* = 5)								
Veraza et al. (37) 2022	Swine forelimbs					24			Closed pressurized systems may provide better edema control.
	Closed pressurized system EVLP (*n* = 9)	15°C–22°C	60 × 20 mmHg pulses/min	Modified Krebs-Henseleit	N/A		11.83% ± 5.26%	No	
	Open EVLP (*n* = 4)						32.86% ± 13.56%		
Tawa et al. (38) 2022	Swine partial hindlimbs	21°C	40 mmHg	Modified Steen with BSA (15%)	Heparin	24		No	PF may provide better results than CF at 24-h preservation with potential improvement in endothelial injury
	Pulsatile (*n* = 3)		Flow adjusted to pressure		Insulin		12.43%		
	Continuous (*n* = 3)		2 L	PEG	Dexamethasone		14.48%		
					Hydrocortisone				
					Penicillin-streptomycin				
Rezaei et al. (39) 2022	Human upper limb (transhumeral)	38°C	90 mmHg	RBC	Heparin	48 or		No	Significantly better MIS than SCS with no significant increase between 6-h marks
	EVLP (*n* = 10)		Flow adjusted to pressure	Fresh frozen plasma	Vancomycin	>115 mmHg	0.4% ± 12.2%		
	SCS (10)		2.5 L	Albumin (%25)	Cefazolin	or >30 mmHg CP			
					Methylprednisolone	or 20% drop O_2_			
						41.6 ± 9.3 h			
Figueroa et al. (40) 2022	Swine forelimbs	38°C	90 mmHg/						
	EVLP-HBOC201		Flow adjusted to pressure	Plasmalyte + HBOC + Albumin	Insulin	22.50 ± 1.71	23.10 ± 3.00	No	HBOC perfusion provides similar results to RBC.
	EVLP-RBC (*n* = 6)		2.5 L	Plasmalyte + RBC + Albumin	Heparin	28.17 ± 7.34	13.18 ± 22.70		Slightly more weight gain with HBOC
	SCS (*n* = 6)				Vancomycin	>115 mmHg			
					Albumin	or >30 mmHg CP			
					Ca gluconate	or 20% drop O_2_			In terms of contractility and histology, no differences were seen.
					Methylprednisolone				
Rohde et al. (41) 2021	Human upper limbs	38°C	No information	RBC	Heparin	41.6 ± 9.4	0.4 ± 12.2%	No	Active metabolism during EVLP
	EVLP (*n* = 7)			Fresh frozen plasma	Vancomycin				Deterioration at the end in histology and metabolism
	SCS (*n* = 7)			Albumin	Cefazolin				
					Methylprednisolone				
Kruit et al. (42) 2021	Swine forelimb	15°C	30 mmHg	UW mp solution	Methylprednisolone	18		Replantation	Histologically worse outcome with EVLP
	EVLP (*n* = 6)		Flow adjusted to pressure				2.7% (19% after rep)	12 h	But neuromuscular function remained similar.
	SCS (*n* = 6)		1 L				1.6% (11.6 after rep)		
Amin et al. (43) 2021	Swine forelimb			Packed RBC [500 ml]	Heparin	6		Reperfusion with blood 4h	Normothermic perfusion at 70 mmHg provided the best results.
	HMP-30 (*n* = 5)	10°C	30 mmHg/FA/1.1 L	500 ml Ringers	Meropenem		8.9% (5.4)	after 6 h NMP 70 (*n* = 5)	
	SNMP-50 (*n* = 5)	28°C	50 mmHg/FA/1.1 L	BSA (%5)	15% glucose		6.3% (3.1)	vs. SCS (*n* = 5)	
	SNMP-70 (*n* = 5)	28°C	70 mmHg/FA/1.1 L		Methylprednisolone		7.4% (1.7)		Upon reperfusion NMP 70 limbs are more stable metabolically vs. SCS.
	NMP-70 (*n* = 5)	38°C	70 mmHg/FA/1.1 L				–0.3% (1.7)		
Said et al. (44) 2020	Swine forelimb	38°C	No target given	Albumin	Vancomycin	>125 mmHg		No	HBOC201 provides superior results compared to SCS.
	EVLP (*n* = 3)		4 L	HBOC-201	Methyl- prednisolone	21.3 ± 2.1 h	25.5 ± 11.7%		
	SCS (*n* = 3)			Glucose	Heparin				
				Electrolytes	Regular insulin				
Haug et al. (45) 2020	Human upper limb	10°C	30 mmHg	Steen	50% Dextrose	24			Hypoxic perfusion with Steen shows better metabolic profile and histology against SCS.
	EVLP (*n* = 3)		Flow adjusted to pressure		methylprednisolone		4,30%	No	
	SCS (*n* = 3)		4 L		heparin		1,40%		
Haug et al. (46) 2020	Swine Forelimbs	10°C	constant flow 20 ml/min		50% Dextrose	12			
	SCS (*n* = 2)				Methylprednisolone		3%	No	Limited number of observations
	Modified Steen (*n* = 2)			Steen	Heparin		25%		Comparable results with cheaper Phodex to Steen
	Phodex (*n* = 2)			Dextran 40 Enriched Phoxillum			36%		
	Phoxillum (*n* = 2)			Phox only			58%		
Fahradyan et al. (47) 2020	Swine Forelimbs	38°C	100 mmHg	albumin	Vancomycin				Paper does not discuss how they manage edema
	EVLP (*n* = 5)		2.5 L	RBC	Methylprednisolone	12	98.72 ± 8.59	No	
	Extended EVLP (*n* = 5)				Heparin	25 h (24–44)	107.28 ± 15.05		
	SCS (*n* = 10)				R insulin				
Gök et al. (48) 2019	Rat hindlimb	30°C–35°C		Steen with Swine RBC (6–9Hb)	Heparin	6			
	1F through femoral (*n* = 5)				Na Bicarbonate		>35%	No	Established parameters were tested against SCS (*n* = 5).
	22G through iliac (*n* = 5)				methylprednisolone		3.1 ± 0.4%		EVLP provided better injury scores in soleus muscle.
					Cefazolin				
					Ca gluconate				
Gök et al. (49) 2019	Rat hindlimbs	10°C	No detail given	5% albumin/HTK	Heparin	6	No information	Transplantation 12 weeks	EVLP histology is similar to immediate replantation.
	Native control (*n* = 5)				Na bicarbonate				Muscle twitch force was higher than SCS.
	Sciatic nerve transection and repair (*n* = 5)				Cefazolin				
	Immediate transplant (*n* = 5)								
	SCS (*n* = 5)								
	EVLP (*n* = 5)								
Krezdorn et al. (50) 2019	Swine forelimb	8°C	30 mmHg	Steen	50% Dextrose	24			Steen-perfused muscle shows better integrity than SCS after reperfusion.
	EVLP (*n* = 4)		Flow adjusted to pressure		Insulin		41%	Replantation	
	SCS (*n* = 4)				Methylprednisolone	4		7 day follow-up	
Kueckelhaus et al. (51) 2017	Swine forelimb	10°C	30 mmHg	Perfadex	Dextrose				Both pre- and post-reperfusion muscle histology scores are better in Perfadex-perfused muscles.
	EVLP (*n* = 3)		Flow adjusted to pressure		Insulin	12	10% ± 2	Replantation	
	SCS (*n* = 4)		5.6 L		Methylprednisolone	4		7 day follow-up	
Duraes et al. (52) 2017	Swine forelimbs (*n* = 18)	35°C	Physiologic pressure	Albumin	Methylprednisolone	12	0.4% mean	No	Simulating physiological conditions with washed RBC provided contraction with good histology for at least 12 h.
	Protocol development study		Flow adjusted to pressure	RBC	Vancomycin		First 13 were colloid only		
	optimized included (*n* = 5)			Glu	Heparin		and colloid + wholeblood		
				Electrolytes	Regular insulin		(% range 17–50)		
Werner et al. (53) 2017	Human forearm	30°C–33°C	<110 mmHg	Plasma based (albumin)	NaHCO3	24	−0.4%,	No	Normal contractility
			Flow adjusted to pressure	Packed RBC (Hb 4–6 g/dl)	Heparin		(−7%–+7%)		Normal histology was preserved.
			250–300 ml.		Dextrose				
					Insulin				
Kueckelhaus et al. (54) 2016	Swine hind limb	10°C	30 mmHg	Perfadex	Methylprednisolone	12	44.06%	No	Histology was better than SCS but 44% weight gain
	EVLP (*n* = 5)		Flow adjusted to pressure		Insulin				Does not discuss
	SCS (*n* = 5)		5.6 L		Dextrose (%50)				
Özer et al. (55) 2016	Swine forelimb	27°C–32°C	Pulsatile 60–80 mmHg	Packed red blood cells	Dextrose or insulin		Numeric value not given	Transplantation	Muscle contractility was preserved.
	EVLP (*n* = 4)			plasma dextran in a ratio of 1:2	as needed	24		12 h follow-up	
	SCS (*n* = 4)								
Özer et al. (56) 2015	Swine forelimb	27°C–32°C	Pulsatile 60–80 mmHg	Packed red blood cells	Dextrose or insulin		Numeric value not given	Transplantation	Near normal single muscle contractility was preserved.
	EVLP (*n* = 4)			plasma dextran in a ratio of 1:2	as needed	24		12 h follow-up	
	SCS (*n* = 4)					6			
Müller et al. (57) 2013	Swine forelimb	32°C	100–150 ml/min	HAES priming	Insulin				Inflammatory profile does not change between groups.
	G1. 6 h ischemia/12 h perfusion (*n* = 7)			autologous blood	methylprednisolone			Replantation	
	G2. 12 h ischemia/w5 h perfusion (*n* = 6)					5	Wet/dry ratio		
	G3. No ischemia/12 h perfusion/replantation/7 days (*n* = 11)					12	No significant changes between groups		*Ex vivo* perfusion is feasible.
	G4. 6 h ischemia/12 h perfusion/replantation/7 days (*n* = 11)					12			
Constantinescu et al. (58) 2011	Swine forelimbs	32°C	100–150 ml/min	Autologous blood	Methylprednisolone				Near normothermic with blood provides minimal weight gain with a good inflammatory profile
	EVLP (*n* = 8)					12	1.32%	No	
	SCS (*n* = 8)								
Tsuchida et al. (59) 2003	Rat hindlimb	25°C	Gravity fed 100 cm	UW	No information	5	No information	Syngeneic	UW perfusion preserves ATP better than ischemia only
	EVLP (*n* = 6)							24 h	
	Without UW perfusion								
Tsuchida et al. (60) 2001	Rat hindlimb	25°C	Gravity fed 40 cm or 100 cm		No information	5	No information	No	UW at 100 cm provides better ATP preservation.
	EVLP-UW (*n* = 8)			UW					
	EVLP-EC (*n* = 8)			EC					
	Control (*n* = 8)								
Yabe et al. (61) 1994	Rabbit hindlimbs	22°C	0.025 mg/g.mn	Perfluorochemical	No information				Better histology with perfusion preservation
	3-h perfusion (*n* = 7)			Oxygen transport Fluid FC-43		3	No information	No	
	6-h perfusion (*n* = 7)					6			
	3-h hypothermia (*n* = 7)								
	6-h hypothermia (*n* = 7)								
	Sham (*n* = 7)								
	Bx (*n* = 7)								
Gordon et al. (62) 1992	Dog hindlimbs	22°C	Pulsatile/0.7 × limb weight	UW	No information	4	No information	No	Perfused limbs preserve ATP better with better histology.
	UW perfusion (*n* = 3)								
	Ischemia (*n* = 3)								
Domingo et al. (63) 1991	Dog hindlimbs	Hypothermia	No info on pressure 725cc	Ringer's lactate	Na Bicarbonate	24	20%–50%	Replantation	Less than 5% of the muscle fibers showed an abnormality when examined, and the lesions were reversible.
	Immediate rep (*n* = 6)			Rheomacrodex					
	EVLP (*n* = 9)								

*Established protocol from Burlage et al. (36).

**Table 2:** *Ex vivo* perfusion of muscle/musculocutaneous flap models.

Author/Year	Animal model and groups	Temp.	PP/Flow/TV	Perfusate base	Additives	Duration (h)	Weight increase (%)	Reperfusion	Remarks
Brouwers et al. (64) 2022	Swine myocutaneous flap	13.5°C	26 ± 3 mmHg		Methylprednisolone	24 h		Yes	Perfusion with HTK solution seemed to result in better histology 7 days post reperfusion compared with UW-MPS.
	UW-MPS (*n* = 2)		Flow adjusted to pressure	UW-MPS	Glucose		−6% and −7%	7 days	
	HTK (*n* = 2)			HTK	Insulin		60%–97%		Markers of muscle damage decreased overall in both perfusion groups.
	Preserved on ice for 4 h (*n* = 2)								
Taeger et al. (65) 2020	Swine rectus abdominis muscle flaps	Room temp.	0.70 (±0.23) ml/h	Colloidal solution (Volulyte 6%)	Glucose	6 h	49.4% (±5.9%)		By using HAES, the results were improved all perfused muscles were able to exert a force response after EFS
	1: Single flush with HAES (*n* = 5)				Calcium phosphate				
	2: perfusion HAES (*n* = 5)				Heparin				
Kruit et al. (66) 2019	Swine rectus abdominis muscle flaps	10°C	Max 30 mmHg	UW-mp	No information	36 h		Yes	PCR analysis of perfused flaps vs. SCS
	Group I: control (*n* = 4) 36 h at 4°C–6°C (static cold storage)		Flow <10 ml/min	HTK		18h	Stable with UW	12-h	IL1B and NFKBIZ expressions up-regulated expression after flap replantation, suggesting activation of the inflammatory response.
	Group II: (*n* = 3) 36 h UW-mp perfusion, fluid temperature of 8°C–10°C								
	Group III: (*n* = 4) control flaps were replanted after 4 h of cold storage						50% HTK		
	Group IV: (*n* = 5) UW-mp perfusion flaps were replanted after 18 h								
	Group V: (*n* = 5) HTK perfusion flaps were replanted after 18 h								
Taeger et al. (67) 2016	Swine rectus abdominis muscle flaps	Room temp.	600 ml/h	Volulyte® 6%	Heparin	6 h	84.0% (±25.1%)	No	Continuous perfusion prevents a rise in Annexin V-positive nuclei.
	1: control (*n* = 5) (flush of Volulyte)								Using a colloidal solution like HES for the formation of edema is reduced compared to simple saline.
	2: iso-oncotic colloid (HES) (Volulyte® 6%) (*n* = 5)(continuous perfusion)								By using HES, the muscles’ ability to react to EFS is somewhat improved.
Taeger et al. (68) 2015	Swine rectus abdominis muscle flaps	Room temp.	600 ml/h	Crystalloid fluid (No detail given)	Heparin	6 h	99.9% (±22.5%)	No	Perfused muscles showed higher ability to exert force compared to nonperfused ones.
	Control (*n* = 5)	(20°C ± 2°C)							These findings were confirmed with Annexin V.
	Crystalloid fluid (*n* = 5)								Perfusion of muscle tissue limits damage compared to nonperfused tissue.
Taeger et al. (69) 2014	Swine rectus abdominis muscle flaps	Room temp.	10 ml/min flow	HTK	Heparin	60 min	No information	No	Expression of Caspase-3 after 60 min. was reduced in all groups compared to the control group.
	I, No treatment = control group (*n* = 4)			Jonosteril	Heparin				All groups (except group III) expressed less HIF-1-a than the control group.
	II, Perfusion with HTK (*n* = 5)								
	III, Singular flush with 10 ml HTK (*n* = 5)								
	IV, Perfusion and oxygenation with Jonosteril (*n* = 5)								
	V, Perfusion and oxygenation with HTK (*n* = 5)								
Dragu et al. (70) 2012	Swine rectus abdominis muscle flaps	Room temp.	600 ml/h	Crystalloid fluid	Heparin	60 min	8.5%	No	During perfusion, additional oxygenation of the perfusion reactor led to different *ex vivo* oxygen tissue saturations, which can be detected by dynamic quenching.
	Experiment I (*n* = 5): perfused with a 291 mosmol/L heparinized crystalloid fluid			Blood					
	Experiment I (*n* = 5): perfused with heparinized blood (500 I.E. heparin/100 ml blood)								
Dragu et al. (71) 2012	Swine rectus abdominis muscle flaps	Room temp.	10 ml/min	Crystalloid fluid	Heparin	60 min	No information	No	The expression of HIF-1 and caspase 3 was increased in both groups without perfusion.
				Blood					HIF-1 and caspase 3 was low during *in vivo* perfusion and extracorporal perfusion with crystalloid fluid.
	I, *in vivo* 5								Heparinized autologous whole blood perfusion shows no protective effect in contrast to the crystalloid.
	II, *ex vivo* 5								The extracorporal perfusion of muscle flaps with crystalloid fluid is a possible protective strategy.
	III, singular heparin flush 5								
	IV, blood perfusion 5								
	V, Jonosteril perfusion 5								
Dragu et al. (72) 2011	Swine rectus abdominis muscle flaps	38	No information	Jonosteril	Heparin	2 h	57 ± 4.5 g before	No	The data of this study indicate that the *ex vivo* perfusion of free muscle flaps is technically feasible.
	Experiment I (*n* = 1): Starting rate of 1 ml/min, flow was raised 1 ml/10 min up to 10 ml/min.						76 ± 15.2 g after		A closed and steady circulation is manageable for a period of up to 2 h.
	Finally, the perfusion rate was increased to 20 ml/min								
	Experiment II–VI (*n* = 5): perfusion 10 ml/min Jonosteril for 1 h								

## The *ex vivo* machine perfusion set-up

**Figure 1:**
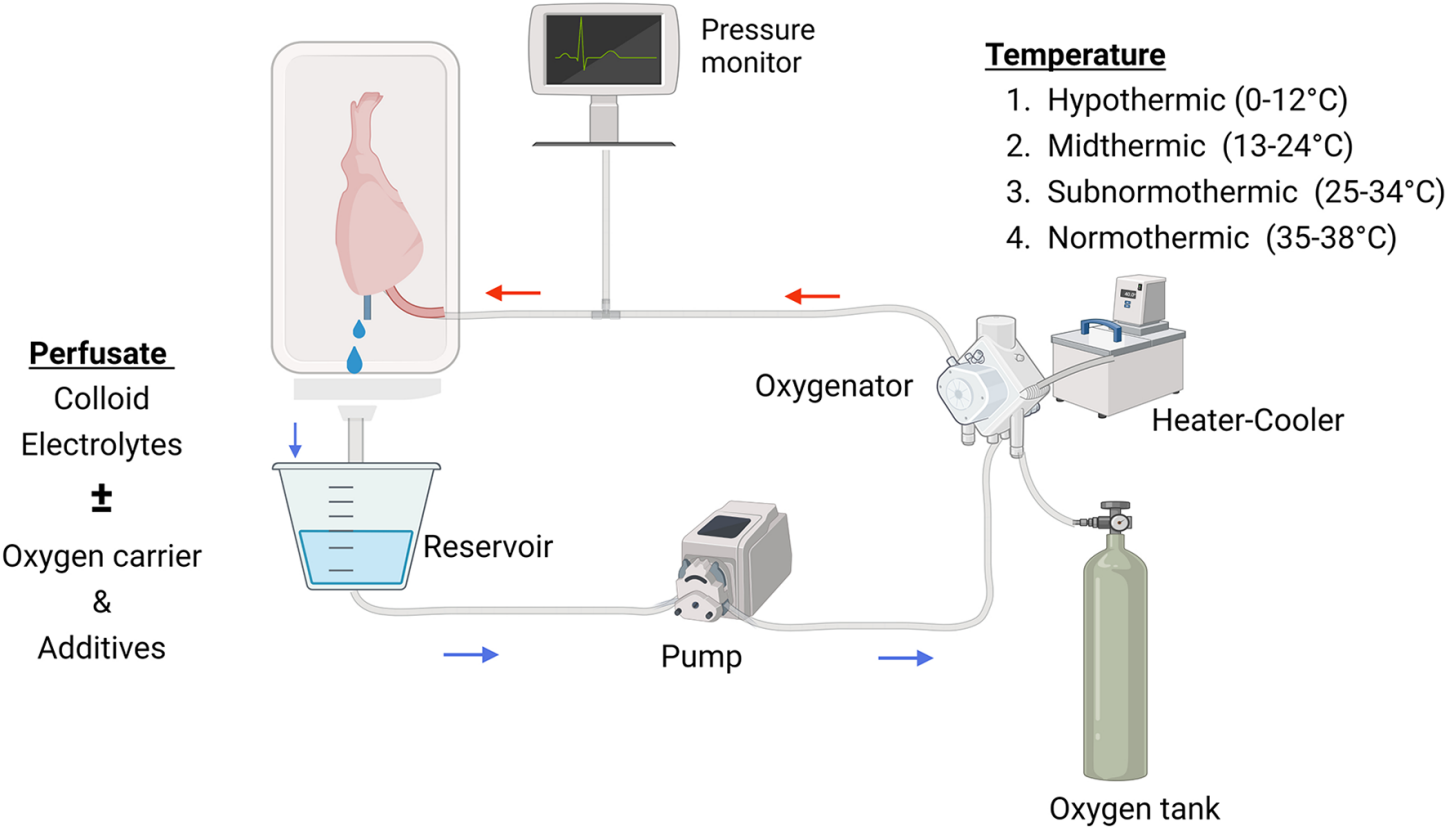
The essentials of *ex vivo* limb perfusion setup. Created with BioRender.com.

An example of a standard *ex vivo* machine perfusion setup is shown in Figure 1. The limb or the composite tissue is procured with its vascular pedicle and the artery is cannulated. The circuit starts with a reservoir containing the perfusate. The perfusate is driven by a pump to a membrane oxygenator attached to a heat exchanger for the delivery of perfusate at the desired temperature. Continuous pressure monitoring is made at the level of the artery. The venous return is gravity-fed back to the reservoir to complete the circuit.

## Temperature

The temperature is the key determinant in the *ex vivo* perfusion of a limb or other composite tissue models as the metabolic activity changes according to temperature. Every 10°C drop in temperature results in about a two-fold decrease in metabolic activity (73). This relationship also impacts the composition of the perfusate and other parameters to meet the demand of the tissue at a given temperature. Currently, there is no consensus regarding the optimal perfusion temperature, and a wide range (4°C–39°C) has been used in experimental models (Tables 1, 2). Perfusion temperatures fall into one of the four categories suggested by Karangwa et al. (74): Hypothermic (0°C–12°C), Mid-thermic (13°C–24°C), Subnormothermic (25°C–34°C), and Normothermic (35°C–38°C). Experiments should be assessed in their temperature context, and this nomenclature will be used to group and review studies in the following sections.

## Perfusate

Since the first attempts at organ preservation with machine perfusion using autologous blood in solid organs, a wide array of commercially available preservation solutions has been developed and experimented with, and custom-made cellular/acellular mixes have also been reported. Thus far, for limb and composite tissue machine perfusion experiments, there have been adaptations from solid organ preservations (Tables 1, 2). In broad terms, a perfusate can be formulated as Colloid + Electrolytes ± Oxygen carrier & Additives. This can be thought of as a mimicry of mammalian blood in which plasma contains proteins that create colloid oncotic pressure along with electrolytes and oxygen is delivered to the tissues via Hemoglobin in red blood cells (RBCs).

### Colloids

Colloids are macromolecules that cannot move through membranes and help to decrease the fluid escape to prevent edema. One of the most common colloids that were used in VCA machine perfusion is dextran 40, a polysaccharide that has been used for plasma volume expansion. It is the colloid used in commercial preservation solutions like Low-potassium dextran (LPD-Perfadex) and Steen solution (LPD solution with albumin) as well as in custom-made RBC-based perfusates. Albumin is the common colloid of choice in RBC-based custom perfusates as well as the aforementioned Steen solution. Pentafraction is a form of hydroxyethyl starch (HAES), which is another polysaccharide, and is the colloid used in UW-MP solution. Another compound used in machine perfusion solutions is polyethylene glycol (PEG), which is used in the enrichment of custom-made perfusates (Tables 1, 2, “Perfusate base” section). The composition of commercial products that have been tested in VCA machine perfusions can be seen in Table 3.

**Table 3:** Commercially available preservation solutions tested in VCA perfusions.

Composition	EC	UW	Steen	Perfadex	HTK (Custodiol)
K^+^	115	125	6	6	10
Na^+^	10	25	138	138	15
Cl^−^	15	20	142	142	22
Ca^2+^	–		0.3	0.3	0.015
Mg^2+^	–	5	0.8	0.8	4
Colloid/Impermeant	–	Pentafraction 50 g/L Lactobionate 100 g/L Raffinose 30	Dextran 40 5 g/L Albumin 7 g/L	Dextran 40 50 g/L	Mannitol 30
Buffer	Phosphate Bicarbonate	Phosphate	Phosphate THAM	Phosphate THAM	Histidine
Antioxidant	–	Glutathione Allopurinol			Mannitol Tryptophan *Α*-ketoglutarate
Glucose	19.5	–	5	5	–
Amino acids					Histidine Tryptophan
Others		Sulfate Adenosine			

Units are given in mmol/L unless otherwise specified.

### Electrolytes

In RBC-based perfusates for electrolytes, crystalloid solutions have been used such as Ringer's Lactate, Plasmalyte (a crystalloid solution that mimics plasma contents closely) (75) or plasma. The mentioned commercial preservation solutions each have different electrolyte compositions, and the important difference is the Na^+^/K^+^ ratio. Solutions that have high Na^+^ are extracellular types while those with high K^+^ are intracellular types. Extracellular solutions mimic the post-ischemic environment and help the recovery of Na^+^/K^+^ -ATPase (76), whereas intracellular solutions compensate for the lack of active transport in an attempt to create cation balance (73) (Table 3).

### Oxygenation and oxygen carriers

Mammalians have an average body temperature of 37.5°C (77), and oxygen delivery is done via hemoglobin. When temperatures are lower, the metabolic rate decreases and the solubility of oxygen increases (78). With respect to this relationship, different oxygenation strategies were derived for different temperature settings.

#### Hypothermic perfusions

Due to the decreased metabolic need under hypothermic conditions, perfusion without an oxygen carrier can be attempted. Direct oxygenation of the perfusate is typically done with a carbogen mixture (%95 O_2_/%5 CO_2_) (45, 50, 51), which is also used in other temperature ranges in both limb and flap models, and the perfusate partial O_2_ pressure is maintained around 300–500 mmHg (45, 46).

#### Mid-thermic and subnormothermic perfusions

For mid-thermic and subnormothermic conditions, perfusion without an oxygen carrier has been attempted by Pendexter (34), Veraza (37), and Kruit (42) in limb models and by Taeger et al. in multiple studies (65, 67, 69) (Tables 1, 2). Most of these studies do not include SCS controls except for that of Kruit et al. in which they report worse outcomes after 18 h of perfusion in histology vs. SCS controls yet preserved muscle contractility after replantation. The effect of adding an oxygen carrier under these conditions (21°C) was tested by Burlage et al. (36) in rat hindlimbs by adding HBOC-201, a hemoglobin-based oxygen carrier polymer (250 kDa) derived from bovine hemoglobin (79) to their custom-made perfusate consisting of BSA, PEG, and muscle cell media. They reported a decrease in weight gain from a mean of 27.3% to 4.9% and better histological outcomes. The idea of preserving a limb at room temperature without any oxygen carrier is appealing because every intervention closing towards normal physiology increases the complexity and the cost of the procedure. It should, however, be noted that due to the high metabolic need of muscle, this idea may only apply for a certain range of temperatures and most probably temperatures approaching the hypothermic range. This would provide only a modest increase in preservation time compared to common SCS conditions.

#### Normothermic perfusions

For normothermic conditions, the need for an oxygen carrier is obvious and reflected in the literature (Tables 1, 2). The most common is the use of RBCs, which is typically arranged to provide a hematocrit range of 10%–15%. Said et al. (44) used HBOC-201 as an oxygen carrier in swine forelimbs and found comparable results to their previous study using RBCs as carriers. Moreover, Figueroa et al. (40) tested HBOC-201 vs. RBC based perfusate in swine forelimbs at normothermic temperatures and reported similar results for histology, although RBCs showed slightly better outcomes in weight increase and compartment pressures (13.18% ± 22.70./29 mmHg ± 15 vs. 23.10% ± 3.00/32 mmHg ± 23). Both groups showed significantly better outcomes than SCS controls.

Hemoglobin-based oxygen carriers (HBOC) are a good prospect in replacing RBCs and should be further studied as an oxygen carrier. RBCs have a significantly lower shelf life and necessitate special storage conditions and are a valuable resource. Moreover, mechanical hemolysis, the need for cross-matching, and the risk of sensitization and transmission of infectious diseases could represent issues. HBOCs, however, are acellular, have low immunogenicity, have a longer shelf life, and can be stored for up to 3 years at room temperature (80, 81).

### Additives

Heparin is a common additive used in most perfusion experiments. This is used in the first batch of the perfusate to remove the residual thrombi that might form after procurement and flushing. Steroids such as methylprednisolone and dexamethasone are also commonly used additives for their effect of decreasing capillary leak and edema, but their relative effect has not been tested in the machine perfusion setting. Antibiotics such as vancomycin, cefazolin, and streptomycin have been used by some groups. Colonization may be a problem, especially for extended perfusion runs, as the perfusion system creates foreign surfaces. Currently, no published data have analyzed bacterial growth during VCA machine perfusions. Further testing will provide insights as to which antibiotics may be needed. Another common additive is the 50% Dextrose-Regular insulin combination. As the amputated limb lacks endocrine control, insulin may be used to enhance glucose uptake to the cells.

To sum up, currently, there is no optimal perfusate composition that has shown consistent results in every given temperature setting and model. Assessments should be made in the context of temperature and procedure. For instance, our group has previously tested the differences between SCS with Heparinized saline, UW, HTK, and Perfadex in an allogeneic rat model and found better results with UW and Perfadex (82). Similar experiments may be attempted especially for hypothermic and mid-thermic perfusion strategies. Studying the limits of mid-thermic perfusion without oxygen carriers can be another good focus due to the relative simplicity of the approach.

## Perfusion pressure & flow

Generally, the flow is adjusted to maintain a pressure goal. The pressure goals vary but for hypothermic perfusions, a 30–40 mmHg perfusion pressure is typically used in all models, including rat, swine, and human. Kueckelhaus et al. (54) report in their pilot studies less endothelial sheer and better structural integrity of the muscle at 30 mmHg compared to 60 mmHg. The same pressure range was also used in a mid-thermic setting by Burlage (36) and Pendexter (34) in rat models. Under the subnormothermic range, Müller et al. (57) and Constantinescu (58) have used 100–150 ml/min with an RBC-based perfusate, which corresponded to 30 mmHg pressure. They reported physiologic pressures resulting in significantly more edema, but numerical data of extremities perfused at physiological pressures were not included in the publication. On the other hand, Özer et al. (55, 56) used pulsatile perfusion. The pulses were driven at 60–80 mmHg with RBC-based perfusate under the same temperatures. In normothermic perfusions, 90 mmHg, which falls into the physiologic level of mean arterial pressure, is commonly used (35, 39, 40, 47).

There are no studies that directly assess different perfusion pressures. However, in a study by Amin et al. (43), four different modalities in swine forelimbs were tested while keeping the perfusate constant (RBC + Albumin based). The first group was hypothermic (10°C) 30 mmHg (HMP-30), the second was subnormothermic (28°C) 50 mmHg (SNMP-50), the third was SNMP-70 mmHg, and the fourth group was normothermic (38°C) at 70 mmHg (NMP-70). Results were reported to be better in terms of histology and weight increase in the NMP-70 group, but they do not discuss differences between the SNMP-50 and SNMP-70 groups. More studies are required to provide better insights in the optimal perfusion pressure for each temperature setting.

## Monitoring the graft during perfusion

*Ex vivo* perfusion platforms allow donor quality assessment during organ preservation. The liver, bile, urea, and coagulation cofactor productions can be monitored (83, 84). In kidney perfusions, urine production can be observed during perfusion, and kidney-specific markers such as NGAL (neutrophil gelatinase-associated lipocalin) can be measured to assess injury in the organ (85). In lungs, ventilation parameters can be analyzed as airway resistance and pulmonary compliance during reperfusion (86). On the other hand, the tissue of interest is not a single specific organ that has a pre-determined, gradable internal function for the continuation of homeostasis in VCA. The overwhelming majority of clinical applications of VCA involve extremity and craniofacial transplantations (87). In limb transplants, the goal is to achieve a viable limb with good motor and sensory function that allows daily activities to be accomplished independently. In craniofacial transplants, the goals are to improve airway stability, mastication, speech, and overall cosmesis depending on the pre-transplant condition of the patient. A common feature in these grafts is the transfer of functional muscle tissue with motor nerve coaptation to the recipient motor nerve ends. Long-term functional outcomes also depend on nerve repair level, regeneration, and rehabilitation (32, 87). In that context, graft monitoring during *ex vivo* perfusion differs from other organs. To document the function of the graft during preservation, assessment of muscle contraction in response to nerve stimulation has been a frequent practice (12 studies in extremity models, 40%) (Table 1). However, it must be emphasized that the depolarization of a muscle fiber at a given time is influenced by factors such as temperature, electrolyte imbalances, and pH (88). A negative response does not mean that the limb is “failing” or a perfusion without any contraction response is worse than a perfusion with contraction. There is no limb specific marker that can be used for every setting; however, regular perfusate gas analysis is important to follow markers such as lactate and potassium. Increased lactate indicates poor tissue oxygenation and a shift to anaerobic respiration in any tissue in the body (89). Potassium is abundant intracellularly (90) and is indicative of cellular damage; however, the models involve cut ends of muscle bodies and some increase is usually observed. Weight increase and compartment pressure are also monitoring modalities that do not require histopathological and metabolic analysis. There is currently no commonly accepted monitoring protocol in *ex vivo* VCA perfusion studies. Studies so far show the following: weight gain, perfusate gas analysis, and histopathological analysis as a common practices in monitoring VCA *ex vivo* perfusions. Compartment pressure, nerve stimulation, and metabolic analysis have emerged as frequent but not universal practices in monitoring VCA *ex-vivo* perfusion.

## Weight gain and when to stop perfusion

A common finding in all limb and VCA perfusions is the weight increase over time due to the fluid escape to the interstitium and the inevitable increase in vascular resistance and compartment pressures. An extremely wide range of weight increases have been reported (0%–99%) (Tables 1, 2).

### Hypothermic perfusions

For hypothermic conditions, Kueckelhaus et al. (54) report a 44.06% mean weight increase in swine hindlimbs after 12 h of perfusion with Perfadex. A subsequent study again by Kueckelhaus et al. (51) reported a 10% mean weight increase in swine forelimbs after 12 h of perfusion with Perfadex. These were replanted with a 7-day follow-up and compared to limbs replanted after 4 h of SCS. The perfusion group had significantly better outcomes in terms of muscle histology after replantation. Krezdorn et al. (50) perfused swine forelimbs for 24 h with Steen solution and observed a 41% mean weight increase. After replantation, perfused limbs showed better histology compared to the 4-h SCS + replantation control group. There was no information on the post-reperfusion weight or compartment examination. Haug et al. (45) reported only a 4.3% weight increase in human limbs when perfused with Steen solution for 24 h with similar perfusion parameters (30 mmHg PP goal, at 10°C).

For flap models, Brouwers et al. (64) perfused swine rectus abdominis myocutaneous flaps for 24 h at 10°C and reported a weight *decrease* in UW perfused flaps (−6% and −7%), whereas HTK perfused flaps had a 97% and a 60% increase in weight. After replantation and follow-up, both groups showed degenerative changes in muscle histology (Table 2).

### Midthermic and subnormothermic perfusions

Kruit et al. (42) perfused swine forelimbs with UW-MP solution for 18 h at mid-thermic temperature (13.5°C) and observed a mean weight gain of −2.7% for perfused limbs vs. +1.6% for SCS controls. The limbs were replanted and followed for 12 h. At the end of the follow-up, perfused limbs had a 19% weight increase, whereas SCS controls had an increase of 11.6%. The perfused limbs showed worse outcomes in histology, but they preserved contractility at the end of reperfusion. Tawa et al. (38) conducted their experiments at 21°C using a modified Steen solution (further enrichment with PEG and albumin) and observed a mean weight increase of 14.48% with continuous flow after 24 h of perfusion in swine partial hindlimbs.

Gök et al. (49) reported a 3.1% weight increase after 6 h in a rat hind limb model at 30°C–35°C using a Steen and RBC mixture. Werner et al. (53) reported a mean 0.4% decrease in weight using a RBC-plasma-based perfusate in human forearms at 30°C–33°C after 24 h of perfusion. Constantinescu et al. (58) reported a 1.32% weight increase after 12 h of perfusion with autologous blood.

Taeger et al. (65, 67, 68), in their multiple studies, reported a wide range of weight gain (49.5%–99%) after 6 h of perfusion in swine rectus abdomins flaps. They reported better tissue preservation with perfusion. However, it should be emphasized that the control groups in these studies were subjected to ischemia at room temperature rather than at 4°C (Table 3).

### Normothermic perfusions

Under normothermic conditions, Duraes et al. (52) reported a 0.54% mean weight increase at 12 h with an RBC + albumin-based perfusate. In the subsequent studies, from the same group (39, 40, 44) they put forth the following as a discontinuation criteria for limb perfusions: (1) Arterial pressure ≥115 mmHg, (2) 20% drop in tissue O_2_ saturation, and (3) Compartment pressure ≥30 mmHg.

In a recent 2023 study, Meyers et al. (35) reported a positive correlation between weight increase, myocyte injury score (MIS), and potassium and lactate levels. There was a negative correlation with muscle contractility under normothermic conditions. In their experiments, they reached a 2% weight increase after 13 ± 5 h, 5% after 15 ± 6 h, 10% after 16 ± 6 h, and 20% after 19 ± 4 h of perfusion. MIS was significantly higher than the baseline at 5% weight increase, and contractility was significantly lower at 20% weight increase, when compared to baseline values. Also, they reported a significant increase in compartment pressures upon the termination of the perfusion compared to SCS-preserved limbs (56.5 mmHg vs. 10.5 mmHg).

## Reperfusion outcomes of *ex vivo* VCA perfusion

The clinical and pathological results after reperfusion are critically important to evaluate the efficacy of machine perfusion. Thus far, 11 studies (36%) in extremity models have included some form of reperfusion. They used blood from unrelated donors (swine) in one study, replantation in five studies (four swine and one canine), and transplantation in five studies (one rat study syngeneic; two rat studies unspecified; two swine studies unspecified). Reperfusion follow-up periods ranged from 4 h to 12 weeks) (Table 1).

Under hypothermic conditions, Kueckelhaus et al. (51) observed higher heart rates, which were accompanied by arrhythmias and a drop in oxygen saturation in static cold stored preserved limb recipients vs. perfused limb (swine, 12 h, 10°C, low-potassium dextran) recipients following replantation. This clinical finding was also accompanied by higher markers of muscle injury (myoglobin, K) in the static cold storage group. Histopathology also showed segmental depletion and vacuolization of the fibers in the cold storage group after 7 days post-reperfusion. Similarly, Krezdorn et al. (50) observed that heart and respiratory rates after replantation were increased in the static cold storage group. There was increased damage in muscle biopsy specimens obtained from animals in the static cold storage group after 7 days when compared with those from animals in the perfusion group (swine, 24 h, 8°C, Steen). Furthermore, Gök et al. (49) observed that at 12 weeks post-transplantation the perfusion group (rat, 6 h, 8°C, HTK) showed similar results to the immediate transplantation group in terms of muscle injury scores and muscle contractility while static cold stored transplantations showed worse outcomes.

Under mid-thermic conditions, Kruit et al. (42) observed higher muscle injury scores in perfused limbs (18 h, 15°C, UW) at 12 h post-replantation when compared to SCS, which was attributed to edema formation during preservation. The mean threshold stimulus for muscle contraction did not differ between cold storage and perfusion groups. Clinical outcomes post-reperfusion were not assessed in this study. In the study from Burlage et al. (36), perfused limbs (rat, 6 h, 21°C, HBOC-201) showed higher transplant survival rates in comparison to SCS controls and were similar to the immediate transplant group at 30 days post-reperfusion.

In the sub-normothermic range, Özer et al. (55) observed similar outcomes in single fiber contractility tests between perfusion (swine, 12 & 24 h, RBC based) 27°C–32°C and normal control groups at 12 h post-transplant. However, SCS-preserved muscle showed a decrease in the contractility test.

Reperfusion after normothermic perfusion was tested by Amin et al. (43) and was achieved by an additional 4 h of perfusion with unrelated donor blood. Perfused limbs were hemodynamically and biochemically stable on reperfusion in comparison to those subjected to SCS, showing lower lactate, normal pH, and less edema.

In flap *ex vivo* perfusion models, two studies have included a reperfusion period (replantation of the flap) using swine rectus abdominis myocutaneous flaps as models (Table 2). Brouwers et al. (64) observed better outcomes of muscle injury in HTK perfused flaps when compared to UW-perfused and cold-stored flaps. Perfusion preservation was under hypothermic conditions for 24 h followed by reperfusion for 7 days. Kruit et al. (66) aimed to analyze the gene expression patterns in perfusion-preserved flaps, using HTK and UW with SCS controls. Their perfusion duration was 18 h followed by a reperfusion period of 12 h while the SCS duration was 4 h. The expression of genes related to ischemia, apoptosis, and inflammation was comparable between the *ex-vivo* perfusion and static cold storage groups.

## Discussion

Four major strategies (Hypothermic, Midthermic, Subnormothermic, and Normothermic) have emerged in VCA preservation as in solid organs with each one having advantages and limitations. Similar strategies have resulted in different outcomes in different studies, and current literature lacks evidence to make conclusions mainly due to the complex nature of these studies. Weight increase and compartment pressure increases are the common consequences of *ex vivo* perfusion, especially after 12 h. The current literature shows that the simulation of physiology (Normothermic/RBC) seems to have better outcomes in terms of edema. However, promising results have also been obtained in other settings even without oxygen carriers.

It must also be noted again that most of the studies so far do not include a reperfusion phase, which is important to fully assess the preservation method (Tables 1, 2, reperfusion outcomes section). To move one step further for clinical translation, the net effect of weight increase on compartment pressures and the possible early post-operative consequences of reperfusing an extremity that already has increased weight/pressures should be thoroughly studied to provide stringent criteria for discontinuation for each model used. After this, optimizing and comparing approaches will be an easier exercise. More studies are needed with reperfusion, especially allogeneic, to better understand the course of machine-preserved limbs against SCS-preserved limbs.

Currently, there is no optimal perfusate composition for limbs and muscle containing composite flaps that has shown consistent results in every given temperature setting and model. Assessments should be made in the context of temperature and procedure. For instance, our group has previously tested the differences between SCS with Heparinized saline, UW, HTK, and Perfadex in an allogeneic rat model and found better results with UW and Perfadex (82). Similar experiments may be attempted especially for hypothermic and mid-thermic perfusion strategies. Studying the limits of mid-thermic perfusion without oxygen carriers can be another good focus due to the relative simplicity of the approach.

Another important aspect of *ex vivo* limb perfusion studies is the sampling of muscle tissues, especially for studies that do not have a reperfusion phase. The weight increase in the proximal part of the limb will not translate into an increase in compartment pressure as the fascial compartment is released during procurement, and over the course of *ex vivo* perfusion this may create differences in viability compared to the distal muscles in the limb confined in their fascial compartments. There have been no studies investigating the possible differences in histopathological or metabolic outcomes of the different levels of the limb. We acknowledge that these experiments are time and resource consuming. However, this will be a good initial step to better understand the effects of weight and compartment pressure increases of *ex vivo* perfusions.

We also acknowledge that the perfusion duration goals in the given examples are arbitrary and designed to demonstrate how much longer an extremity or VCA can be preserved by using machine perfusion. In a future clinical scenario completing the whole procedure “as soon as possible” will remain a goal both for transplantation and replantation cases. Nevertheless, *ex vivo* perfusion as a method of preservation for VCA is an exciting field of research with a high potential for clinical translation.
